# Linguistic summarization of visual attention and developmental functioning of young children with autism spectrum disorder

**DOI:** 10.1007/s13755-024-00297-4

**Published:** 2024-07-16

**Authors:** Demet Öztürk, Sena Aydoğan, İbrahim Kök, Işık Akın Bülbül, Selda Özdemir, Suat Özdemir, Diyar Akay

**Affiliations:** 1 Department of Industrial Engineering, Gazi University, Ankara, Turkey; 2 Department of Computer Engineering, Pamukkale University, Denizli, Turkey; 3 Department of Special Education, Gazi University, Ankara, Turkey; 4 Department of Special Education, Hacettepe University, Ankara, Turkey; 5 Department of Computer Engineering, Hacettepe University, Ankara, Turkey; 6 Department of Industrial Engineering, Hacettepe University, Ankara, Turkey

**Keywords:** Linguistic summarization, Autism spectrum disorder, Eye-tracking, Bayley, Fuzzy logic

## Abstract

Diagnosing autism spectrum disorder (ASD) in children poses significant challenges due to its complex nature and impact on social communication development. While numerous data analytics techniques have been proposed for ASD evaluation, the process remains time-consuming and lacks clarity. Eye tracking (ET) data has emerged as a valuable resource for ASD risk assessment, yet existing literature predominantly focuses on predictive methods rather than descriptive techniques that offer human-friendly insights. Interpretation of ET data and Bayley scales, a widely used assessment tool, is challenging for ASD assessment of children. It should be understood clearly to perform better analytic tasks on ASD screening. Therefore, this study addresses this gap by employing linguistic summarization techniques to generate easily understandable summaries from raw ET data and Bayley scales. By integrating ET data and Bayley scores, the study aims to improve the identification of children with ASD from typically developing children (TD). Notably, this research represents one of the pioneering efforts to linguistically summarize ET data alongside Bayley scales, presenting comparative results between children with ASD and TD. Through linguistic summarization, this study facilitates the creation of simple, natural language statements, offering a first and unique approach to enhance ASD screening and contribute to our understanding of neurodevelopmental disorders.

## Introduction

Autism spectrum disorder (ASD) is a complex developmental disorder characterized by persistent difficulties with social communication, limited interests, and repetitive behavior. It is classified as a neurodevelopmental disorder in the Diagnostic and Statistical Manual of Mental Disorders (DSM-V) [1]. According to the DSM-V manual, the diagnostic criteria for ASD include persistent deficits in social communication and Social Interaction (SI) across multiple contexts. Symptoms include abnormal social approaches, failures in reciprocal conversation, reduced sharing of interests and emotions [2]. Other indicators are an inability to initiate or respond to SIs, deficits in nonverbal communicative behaviors used for SI, and abnormalities in eye contact [3]. Because ASD is a spectrum condition, it presents differently in each individual and challenges in social communication, repetitive behaviours, and sensory sensitivities typically emerge in childhood and last throughout a person's life. Symptoms first manifest in childhood, but it takes 2–3 years—typically until the kid is 4 years old—for an ASD diagnosis to be made. Autism detection is a challenging undertaking that takes time and effort to improve instances. Numerous behavioural and physiological strategies have been employed to reliably and successfully identify autism in children in the early stages of the disorder. Along with informing scientific research centers about the need for appropriate solutions and treatments, predictive indicators are also required to provide parents with early information about their children's behaviour, physiological status, and course [4]. Clinical assessments, developmental histories, and behavioural observations are all critical factors in the diagnosis of ASD. Nonetheless, there has recently been an increase in interest in using data analytics methods, including eye tracking, to help diagnose and comprehend ASD. Although eye tracking and other data analytics methods have the potential to further our knowledge of ASD and enhance diagnostic procedures, it's critical to acknowledge that they are only one component of the diagnostic picture. To give the most thorough understanding of ASD and drive individualized therapies and support measures, integrated approaches incorporating several assessment methods, such as behavioural observations, standardized tests, and neuroimaging techniques, are likely to be used [5]. Early identification of ASD is essential to ensure that children can access specialized evidence-based interventions [6]. Infants at risk for ASD may display different eye gaze patterns than TD infants [7]. Identifying these differences can help in early detection and intervention [8]. Individuals with ASD often have difficulty understanding and responding to social signals, and eye tracking can quantify and analyze these challenges [9]. Recent improvements in hardware and software technology have led to a rise in the creation of eye-tracking applications. These days, wearable, low-cost, and inconspicuous gadgets that generate data that can be quickly evaluated with specialized software have replaced bulky, costly, and time-consuming equipment [10]. Currently, behavioural, historical, parent-report, and interview assessments—all subjective, labor-intensive, and time-consuming—are the primary tools used to diagnose ASD. The screening and diagnosis of ASD are limited by the absence of reliable procedures for assessment. Eye movements have shown promise as biomarkers in neuropsychiatric and cognitive diseases, as well as in ASD, since they provide a window into behaviour, cognition, and decision-making. ET technology could be used to objectively quantify the deficits that people with ASD have in detecting social scenes, making and maintaining eye contact, and recognizing facial information, according to previous results. Large-scale temporal and spatial sequence data, as well as a variety of visual attention variables, are produced by eye-tracking evaluations. Machine learning (ML) algorithms can use these data to classify diseases and support clinical decisions [11]. Linguistic summarization as a descriptive machine learning technique can help understand features affecting this classification and clinical decisions accordingly.

The Bayley Scales of Infant and Toddler Development [12] (Bayley Scales) is a widely used assessment tool to evaluate the developmental progress of infants and toddlers [13]. The Bayley assessment provides comprehensive data on a child's cognitive, language, and motor skills [14]. For decades, the Bayley Scales has been the most widely used objective measure of early developmental delay in clinical and research settings [15]. However, it is a challenging point for non-experts working through ET data or Bayley scales of children to figure out the difference between children with ASD and TD children. In this case, linguistic summarization is crucial and valuable in providing insights from the ET data and Bayley scores of children in natural language. Linguistic summaries from the data can help families better understand their child's strengths and areas for improvement and pay attention.

In this study, we developed linguistic summaries, which are simple to comprehend, to identify differences between children with ASD and TD in natural language form by using the ET data of the project with the Bayley scores of children, which are collected at Gazi University, Learning Development and Education Research Center [16]. Using ET data from this project [16] linguistic summaries for children were created to compare ET characteristics of children [17]. However, that study is limited regarding the data set and techniques. Accordingly, this study aims to extract comprehensive and beneficial information on differences between children from structured data and provide insights with sentences that are useful and straightforward to understand for professionals, clinicians, and researchers who are working on ASD screening. This study represents a fresh method in the field by integrating Bayley scores data, linguistic summarization, and ET data in a way never done before. It offers a thorough grasp of child development and is the first of its type to mix these various sources and methodologies. It creates new chances for comprehensive evaluation and intervention techniques to support children's best possible developmental findings.

In the second section, the background of ET studies and Bayley assessment in ASD screening are provided. In the third section, linguistic summarization techniques are explained. Application and results are in the fourth section. Limitations and Future Directions and Conclusion are in the fifth and sixth sections, respectively.

## Related background

**Fig. 1:**
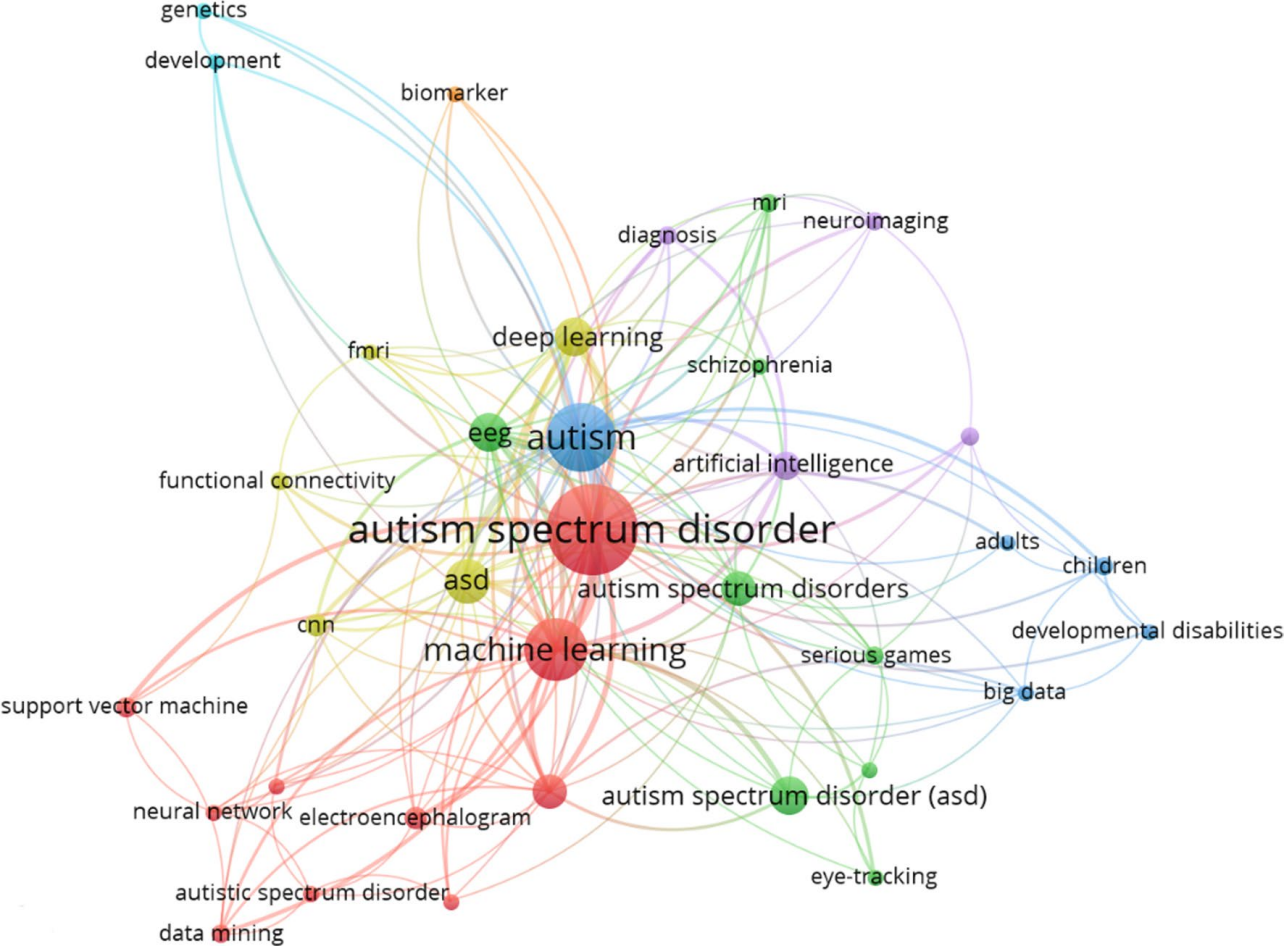
Literature network map

ASD has long been associated with data analytics studies. Therefore, the literature review aimed to uncover existing studies on data analytics for ASD screening. Using VOSviewer, which is a software tool for visualizing bibliometric maps [18], version 1.6.19 [19], co-occurrence analysis with keywords of the bibliographic data of the articles found in the Scopus database was performed, and the network map is given in Fig. 1. The search was done with the keyword "autism spectrum disorder" and refined with the "data analytics" keyword. English was selected as the language criterion. Article, conference paper, review, book chapter, book and conference review document types were included. A year filter wasn’t applied, and 451 sources were used to create a literature map.

It is easily understandable from Fig. 1 that ET data is commonly used in the ASD research area. It is also seen that many different artificial intelligence (AI) techniques have been used for data mining or ML for classification tasks. When we look into the last 5 years (2019–2023) of research, it is shown that ET has been used in several data analysis studies for ASD. These studies revealed that feature extraction from ET data is challenging, and several methods have been used as Rahman et al.’s [20] suggested classification-based method. Integrating ET with other screening data, such as electroencephalography (EEG) and ML, is widely used for ASD screening and detection in children [21–28]. A classification model for ASD prediction is created by combining multiple types of data collected over 3 years, including clinical assessment, neuroimaging and gene mutation data [29]. ET data-based prediction model reliably represents expert hand-coded social visual behavior scores [30]. Multivariate time series analysis is proposed to analyze the temporal relationship between 3D head position angles and object displacement, and its validity is demonstrated by applying it to a video dataset [31]. Classification tasks for ASD with different data sets showed high accuracy with Support Vector Machines [32], without using any imaging device data with Artificial Neural Networks algorithm [33], and with synthetic data [34]. Recently, high accuracy has been achieved by using AlexNet [35]. However, methods like AlexNet and LSTM incur resource and computation overhead when many datasets are on a single node. Therefore, Lakhan et al. [36], presented the federated learning-enabled distributed fog cloud computing environment to improve the overhead of a single node for multimodal datasets. In the field of medicine, early screening for ASD is crucial. Currently, computer-aided diagnostic technologies are utilized in conjunction with AI to enhance the process of diagnosing autism [37]. While the analysis of ET data has been a focal point in data analysis of ASD screening, a significant gap exists: The absence of Bayley scores in data analytics-based research endeavours. Addressing this gap, linguistic summarization integrating ET data with Bayley scores emerges as a critical approach. The significance lies in its potential to generate clear and comprehensible language-based outputs, serving as a valuable resource for experts and non-experts. Recently, the importance of using multiple sources in diagnosis has been emphasized in many studies. For this reason, developing innovative diagnostic tools is vital from a methodological and technological perspective.

Certain crucial behavioural traits are highly predictive of autism and its severity, even when clinical and physiological traits are not recognized at an early age. Because ET technology is quick, affordable, simple to analyze, and appropriate for all age groups, it is one of the most significant and promising indications for ASD. Creating, following, and capturing points to compute eye movement through these points is known as eye movement tracking. As biomarkers of ASD, numerous studies have shown that eye movements have a significant impact on how people respond to verbal and visual signals. Additionally, a few studies have demonstrated a link between clinical testing and early diagnosis of ASD by eye movement tracking. Genetic factors contribute to some of these relationships. Furthermore, ET diagnosis is helpful in the short run for identifying children with ASD [4]. The significance lies in its potential to generate clear and comprehensible language-based outputs, serving as a valuable resource for experts and non-experts. ET is a sensitive method for analyzing behavior and adjusting vision to handle various visual stimuli. In the past, studies have concentrated on using ET to diagnose ASD as well as the biological and behavioral patterns of eye movement, particularly in children who have experienced a variety of developmental disorders, including ASD. ET technology has numerous benefits as a biomarker for diagnosing autism in children. First, it makes eye-tracking ET easier for young children, which means autism risks can be identified early. Second, various information from ET data is utilized to identify abnormal visual focus or biomarkers. Last, ET technology provides a simple and direct assessment connected to the diagnostic instruments for ASD [4]. Combining complex data with easily understandable summaries can enhance the efficacy and accessibility of ASD screening procedures. Knowing that in the study [17] only one set of ET data is used, fundamental evaluation methods are applied and interpretability is not implemented; in this study, we focused on linguistic summarization of ET data with Bayley scores of children, which provide more comprehensive results to make screening for autism more accessible to everyone. Combining ET data and Bayley scores with clear language could change the understandability of ASD and help clinical research on children with ASD.

## Linguistic summarization techniques

Business critical or specific decisions are taken based on data. This makes the analysis and practical interpretation of data important. Considering that the volume and variety of data are rising every day, it is clear that data should be summarized to have valuable insights. Data analytics includes descriptive and predictive analytics [38]. Predictive analytics is related to estimating what is expected in the future. Descriptive analytics identifies patterns using statistical measures based on historical and current data. Descriptive analytics includes linguistic summarization as well as statistical summarization. Summary statistics is one of the ways to create aggregated results that provide a concise overview of the data's distribution, central tendencies, and variability. Therefore, it can be used in different areas such as finance, marketing, and health. In health, enhanced comprehension of medical data may be possible through the rapidly developing and highly significant field of data analytics in the healthcare industry [39]. But, medical data is challenging to interpret with summary statistics. Hence, linguistic summarization makes it easy to interpret and use the data. Furthermore, linguistic summarization reveals the features that affect classification techniques and improves data comprehension. While only statistical summarization is used, the information obtained is limited and may not be easily understood by people. A key constraint is the intricacy of statistical approaches and their dependence on mathematical expressions, which may prove difficult for individuals lacking expertise to understand. Furthermore, quantitative measures are frequently the focus of traditional statistical research, which may not adequately represent the intricacies of real-world occurrences. Traditional statistical studies might also have limitations due to their strict experimental designs and dependence on preconceived hypotheses [40]. Given these drawbacks, it is imperative to supplement conventional statistical techniques with alternative research methods, such as linguistic summarization, which shows promise in addressing some of the drawbacks of conventional statistical studies in terms of delivering understandable knowledge.

Linguistic summarization uses natural language processing methods in conjunction with underlying mathematical formulas to provide accessible findings to non-expert humans. For this reason, Yager proposed the linguistic summarization method [41], which allows data to be summarized in a way that humans can easily understand with the help of natural language and fuzzy sets. Linguistic summarization applications in data analysis involve generating concise and human-readable summaries of large and complex datasets using natural language [42]. Linguistic summarization bridges raw data and meaningful insights, allowing decision-makers to make informed choices based on the summarized information [43]. It has been applied in many different business domains [44]. These summaries enable executives and managers to make data-driven decisions without delving into the intricacies of the underlying data [45]. Furthermore, summarizing medical data can assist healthcare professionals in making accurate diagnoses and identifying trends in patient outcomes [46]. Fuzzy-based approaches are widely used in medical literature, especially in diagnosing [47–50] and detecting well-known diseases [51–54].

The theory of fuzzy sets serves as the foundation for linguistic data summarization. Fuzzy sets are mathematical sets that allow values to have membership degrees between 0 and 1. Lotfi Zadeh introduced the concept of fuzzy sets in 1965 [55]. In Zadeh's definition [55], a fuzzy set $A$ in a universe of discourse $X$ is characterized by a membership function ${\mu }_{A}$ that assigns to each element $x$ in $X$ a degree of membership ${\mu }_{A}(x)$ in the interval [0,1]. The membership function ${\mu }_{A}(x)$ represents the degree to which x belongs to A. When ${\mu }_{A}(x)=0$, x is not a member of $A$, and when ${\mu }_{A}(x)=1$, $x$ is a full member of A. For values of ${\mu }_{A}(x)$ between 0 and 1, $x$ is a partial member of $A$, meaning it belongs to $A$ to some degree. A fuzzy set allows a member to have a partial degree of membership, and this partial degree membership can be mapped into a function [56]. Assume $A$ is a fuzzy set, and element $x$ is a member of this fuzzy set $A$. This mapping can be denoted as Eq. (1). When the universe of discourse $X$ is discrete and finite, this mapping can be expressed as Eq. (2). When the universe $X$ is continuous and infinite, the fuzzy set $A$ can be represented as Eq. (3). The symbols "$\sum$, $\int$, $/$" and "$+$" used in the fuzzy set definition do not have any algebraic meaning. If the set is continuous, it is represented by $\int$. If the set is discrete, it is characterized by $\sum$.

1 $$\mu_{A} \left( x \right) \to \left[ {0,1} \right],\quad \forall x \in X$$

2 $$A = \frac{{\mu_{A} \left( {x_{1} } \right)}}{{x_{1} }} + \frac{{\mu_{A} \left( {x_{2} } \right)}}{{x_{2} }} + \cdots + \frac{{\mu_{A} \left( {x_{n} } \right)}}{{x_{n} }} = \mathop \sum \limits_{i = 1}^{n} \frac{{\mu_{A} \left( {x_{i} } \right)}}{{x_{i} }}$$

3 $$A = \left\{ {\smallint \frac{{\mu_{A} \left( x \right)}}{x} } \right\},\quad \forall x \in X$$

$\alpha$ cuts are used to convert fuzzy sets to crisp sets. If the membership degree of an element to the fuzzy set *A* is greater than or equal to $\alpha$, the membership degree of this element to the crisp set indicated by ${A}_{\alpha}$ takes the value 1. If the membership degree of an element to the *A* fuzzy set is only greater than α, the membership degree of this element to ${A}_{{\alpha }^{+}}$ crisp set takes the value 1 [57]. With $\alpha \in [\text{0,1}$], the representations of $\alpha$ cut and ${\alpha }^{+}$ cut are given in Eqs. (4) and (5).

4 $$A_{\alpha } = \left\{ {x \in X{|}\mu_{A} \left( x \right) \ge \alpha } \right\}$$

5 $$A{ }_{{\alpha^{ + } }} = \left\{ {x \in X{|}\mu_{A} \left( x \right) > \alpha } \right\}$$

A class of objects known as a fuzzy set has a range of membership degrees. One way to identify such a set is via its membership function, which gives each object a membership degree between zero and one [55]. The definition of membership functions for fuzzy sets can be done in various ways. One of these techniques, fuzzy c-means clustering (FCM), was created by Bezdek et al. [58]. The algorithm finds each cluster's centers and each set member's membership degree at the end of this process. Discrete, triangular, and trapezoidal functions are typically utilized despite other membership functions in the literature because of the low processing cost [59, 60].

The four elements of a linguistic summary are (i) a linguistic quantifier $$Q$$ labeled with a fuzzy set, (ii) a linguistic summarizer $$S$$ labeled with a fuzzy set, (iii) a linguistic pre-summarizer $${S}_{g}$$ labeled with a fuzzy set, and (iv) the truth degree of the summary $$T$$, which takes a value in [0,1], and expresses the degree to which the data supports the generated summary [61]. Table 1 shows the symbols used in the linguistic summarization process and their explanations. Zadeh [62] presented two sentence structures with a quantity meaning. Type-I quantified sentences are like "$$Q Y{\prime}s are/have S. [T].$$" An example of this structure is "$$Most of the children have high animation dwell time [0.80]$$". Accordingly, "most", "children", "high animation dwell time" and [0.80] refer to quantifier $$Q$$, subjects $$Y$$, summarizer $$S$$, and truth degree $$T$$ in the type-I sentence, respectively. Type-II quantified sentences are Zadeh's other suggested sentence form [62]. The sentence is written as "$$Q {S}_{g} Y s are/have S. [T].$$" If the cognitive feature of children is also in the dataset, this structure allows for the example "Most of the children behind by age have high animation dwell time [0.70]" to be used. "Most", "behind by age", "children", "high animation dwell time", and [0.70] refer to quantifier $$Q$$, pre-summarizer $${S}_{g}$$, subjects $$Y$$, summarizer $$S$$, and truth degree $$T$$ in the type-II sentence, respectively. These two sorts of sentences are based on the absolute and relative quantifiers provided by Zadeh [62]. "About three" and “at least half" are the examples of absolute and relative quantifiers, respectively. The combination of the followings gives the total number of sentences: the number of quantifiers, the number of summarizers, and (if it exists) the number of pre-summarizers. The most insightful and practical sentence reveals the highest degree of truth [63].

**Table 1:** List of the symbols that are utilized in linguistic summarization

Symbol	Description	Example
${\mathbb{D}}$	Database	Children database
*Y*	The set of all objects in the database	Children with ASD
${y}_{M}$	The ${m}{\text{th}}$ object in the database	${m}{\text{th}}$ child
${v}_{k}$	The ${k}{\text{th}}$ attribute	Animation Dwell Time
${X}_{k}$	The domain of ${v}_{k}$	[590–13051] Animation Dwell Time
${V}_{k}^{m}$	A value of the ${k}{\text{th}}$ attribute for ${m}{\text{th}}$ object	Animation Dwell Time of *m*th child
${d}_{m}$	A complete record of ${y}_{m}$ with values assigned to all attributes	[3965 Animation Net Dwell Time, …]
${S}_{k}$	Summarizer	Medium, high,…
$Q$	Quantifier	Most, few…
${S}_{g}$	Pre-summarizer	Ahead by age, behind by age…
$T$	Degree of truth	0.95

The most important part of the generation of linguistic summaries is the evaluation of the sentences. Summaries are evaluated by calculating the degree of truth. The degree of truth in linguistic summarization refers to the level of confidence that may be placed in the information presented in the summary. A summary with a high truth degree is more reliable and trustworthy than a low one. The degree of truth is important in linguistic summarization because it influences decision-making. Decision-makers depend on summaries to help them make well-informed decisions quickly. A biased or untrustworthy description could sway the decision in the incorrect direction. The truth degree evaluation is a crucial step in the linguistic summarization process since it influences how valuable the summary is [64]. The way to compute the degree of truth is classified into two groups according to the type of cardinality: scalar cardinality-based methods and fuzzy cardinality-based methods. First, Zadeh [62], Yager [65], Bosc, and Lietard [66] proposed using scalar cardinality to compute the degree of truth. The scalar cardinality-based methods have been widely used in the application of linguistic summarization as their computational cost is very low [67]. Scalar cardinality-based methods for Type-I sentences are scalar cardinality-based truth degree calculation by Zadeh [62], truth degree based on Ordered Weighted Averaging (OWA) operator [65], and Choquet integral-based truth degree [66, 68]. Evaluating Type-II quantifier sentences is more complex than assessing Type-I sentences. Therefore, the number of truth degrees suggested for evaluating type-II quantifier sentences is less than those recommended for evaluating type-I quantifier sentences. Scalar cardinality-based methods for Type-II sentences are scalar cardinality-based truth degree calculation by Zadeh [62] and truth degree based on the OWA operator [65]. Methods proposed by Zadeh serve as the foundation for the scalar cardinality-based truth degree calculation methods [69]. The calculation method for type I quantified sentence is given in Eq. (6), and the calculation method for type II quantified sentence is provided in Eq. (7) as $Q$: linguistic quantifier (e.g., most, about three, etc.), $Y$: (*m* = 1,…,*M*) subjects, $S$: summarizer, $T$: truth degree [0,1], $\mu$: membership function, ${d}_{m}$: the value of the feature d of the ${m}{\text{th}}$ object, ${S}_{g}$ pre-summarizer, ${v}_{g}^{m}$: the value of the feature g of the ${m}{\text{th}}$ object.

6 $${\text{T}} = \mu_{Q} \left( {\frac{{\mathop \sum \nolimits_{m = 1}^{M} \mu_{s} \left( {d_{m} } \right)}}{R}} \right),\quad R = \left\{ {\begin{array}{*{20}c} {M,} & {Relative\;quantifier} \\ {1,} & {Absolute\;quantifier} \\ \end{array} } \right.$$

7 $${\text{T}} = \mu_{Q} \left( {\frac{{\mathop \sum \nolimits_{m = 1}^{M} \left( {\mu_{{S_{g} }} \left( {v_{g}^{m} } \right) \otimes \mu_{s} \left( {d_{m} } \right)} \right)}}{{\mathop \sum \nolimits_{m = 1}^{M} \mu_{{S_{g} }} \left( {v_{g}^{m} } \right)}}} \right)$$

If there is more than one summarizer in the quantified sentence created, their intersection is obtained with the t-norm operator $\otimes$ and included in the truth degree calculation [63]. Let's ${A }_{1}$ and ${A}_{2}$ are fuzzy sets defined in $X$ universal set, and the membership functions of these sets are ${\mu }_{{A}_{1}}\left(x\right)$ ve${\mu }_{{A}_{2}}\left(x\right)$.${A}_{1} and{ A }_{2}$, while intersection of sets are${A}_{1} \cap {A}_{2}$; membership function${\mu }_{{A}_{1}\cap {A}_{2}}\left(x\right)$, $\otimes :\left[\text{0,1}\right]x\left[\text{0,1}\right]\to [\text{0,1}]$ is defined in Eq. (8). [69].

8 $$\mu_{{A_{1} \cap A_{2} }} \left( x \right) = \otimes \left( {\mu_{{A_{1} }} \left( x \right),\mu_{{A_{2} }} \left( x \right)} \right){ }\forall x \in X$$

Methods based on scalar cardinality are advantageous because their computational costs are very low. However, using scalar cardinality when calculating the truth degree may produce inconsistent results in some cases because a large number of small membership degrees will overwhelm a small number of large membership degrees [69]. Therefore, scalar cardinality-based methods cannot indicate changes in small truth degrees. The literature proposes fuzzy cardinality-based methods to evaluate sentences [70]. Semi-fuzzy quantifier-based methods are the more general form of fuzzy cardinality-based methods, which can assess sentences generated with a fuzzy quantifier. In cases where features are expressed with fuzzy sets, quantified sentences can be modeled with semi-fuzzy quantifiers, which is the midpoint between a classical quantifier and a fuzzy quantifier [71]. The semi-fuzzy quantifier only accepts the exact argument as a classical quantifier, but its degree of truth is equal to a value in [0,1] as a fuzzy quantifier [72]. Semi-fuzzy quantifiers are much more intuitive and easier to define than fuzzy quantifiers, but they do not resolve the problem of evaluating fuzzy quantified sentences. Therefore, different fuzzification mechanisms have been proposed [73] enabling us to transform semi-fuzzy quantifiers into fuzzy quantifiers. These are the *M* mechanism [74] and the probabilistic *F*^*I*^ mechanism [75]. *F*.^*I*^ mechanism, which is used in this application, is defined below. Suppose E is some set, **I** = [0,1], $X\epsilon \widetilde{\wp }(E)$ and $\gamma \epsilon \mathbf{I}$**.** ${X}_{\gamma }^{min}$, ${X}_{\gamma }^{max}$ $\epsilon \wp (E)$ are defined by Eqs. (9) and (10)

9 $$X_{\gamma }^{min} = \left\{ {\begin{array}{*{20}c} {X_{ > 1/2} } & {\gamma = 0} \\ {X_{{ \ge \frac{1}{2} + \frac{1}{2}\gamma }} } & {\gamma > 0} \\ \end{array} } \right.$$

10 $$X_{\gamma }^{max} = \left\{ {\begin{array}{*{20}c} {X_{ \ge 1/2} } & {\gamma = 0} \\ {X_{{ > \frac{1}{2} - \frac{1}{2}\gamma }} } & {\gamma > 0} \\ \end{array} } \right.$$

where ${X}_{\ge \alpha }=\left\{ e \epsilon E : {\mu }_{x}\left(e\right) \ge \alpha \right\}$ is α cut and ${X}_{>\alpha }=\left\{ e \epsilon E : {\mu }_{x}\left(e\right)> \alpha \right\}$ is strict α cut. The fuzzy median med_1/2_: **I** × **I** ➝ **I** is defined by Eq. (11). The generalized fuzzy median m_1/2_: $\wp \left(\mathbf{I}\right) \to \mathbf{I}$ is defined by Eq. (12) where *inf* as the biggest lower bound and *sup* as the smallest upper bound. Fuzzy quantifier ${Q}_{\gamma }: \widetilde{\wp }{(Y)}^{K}\to \mathbf{I}$ is defined by Eq. (13) for all semi-fuzzy quantifiers$Q$: $\widetilde{\wp }{(E)}^{s}$ → I.

11 $$med_{\frac{1}{2}} \left( {u_{1} ,u_{2} } \right)\left\{ \begin{gathered} \begin{array}{*{20}c} {\min \left( {u_{1} ,u_{2} } \right)} & {min\left( {u_{1} ,u_{2} } \right) > 1/2} \\ \end{array} \hfill \\ \begin{array}{*{20}c} {\max \left( {u_{1} ,u_{2} } \right)} & {max\left( {u_{1} ,u_{2} } \right) < 1/2} \\ \end{array} \hfill \\ \begin{array}{*{20}c} \frac{1}{2} & {otherwise} \\ \end{array} \hfill \\ \end{gathered} \right.$$

12 $$m_{\frac{1}{2}} \left( X \right) = med_{\frac{1}{2}} \left( {\inf X ,\sup X} \right) for all X \epsilon \wp \left( {\mathbf{I}} \right)$$

13 $$Q_{\gamma } \left( {X_{1} ,X_{2.} \ldots ,X_{k} } \right) = m_{\frac{1}{2}} \left\{ {{\text{Q}}\left( {Y_{1} ,Y_{2.} \ldots ,Y_{s} } \right) : (X_{i} )_{\gamma }^{min} \subseteq Y_{i} \subseteq (X_{i} )_{\gamma }^{max} } \right\}$$

Accordingly, let $Y$ be the linguistic universe, ${S}_{1}, {S}_{2},\dots ,{S}_{k}\epsilon \widetilde{\wp }(Y)$ are linguistic summaries of fuzzy sets defined in the universal set $Y$,$\gamma \epsilon [\text{0,1}]$. The probabilistic mechanism ${F}^{I}$ is defined as Eq. (14) where${S}_{k}$, k = 1,…,K $\epsilon \widetilde{\wp }(Y)$ are fuzzy sets; ${\left({S}_{k}\right)}_{\ge {\alpha }_{k}}$ is α – cut level ${\alpha }_{k}$ of${S}_{k}$; and $Q$ is a semi-fuzzy quantifier of arity K.

14 $$F^{I} \left( Q \right)\left( {S_{1} , \ldots ,S_{k} } \right) = \mathop \smallint \limits_{0}^{1} \ldots \mathop \smallint \limits_{0}^{1} Q\left( {\left( {S_{1} } \right)_{{ \ge \alpha_{1} }} , \ldots ,\left( {S_{k} } \right)_{{ \ge \alpha_{k} }} } \right)d\alpha_{1} \ldots d\alpha_{k}$$

Based on ${F}^{I}$ mechanism, we may calculate the degree of truth in the sentence "Almost all children with ASD behind by age have high animation net dwell time." Let's behind by age = A, high animation net dwell time = B, almost all = Q and

$$A = \left\{ {\frac{0.8}{{e_{1} }},\frac{0.9}{{e_{2} }},\frac{1}{{e_{3} }},\frac{0.2}{{e_{4} }}} \right\},\;B = \left\{ {\frac{1}{{e_{1} }},\frac{0.8}{{e_{2} }},\frac{0.3}{{e_{3} }},\frac{0.1}{{e_{4} }}} \right\}$$

$${\mathbf{almostall}}_{E} \left( {X_{1} ,X_{2} } \right) = \left\{ {\begin{array}{*{20}c} {max\left\{ {2\left( {\frac{{\left| {X_{1} \cap X_{2} } \right|}}{{\left| {X_{1} } \right|}}} \right) - 1,0} \right\}} & {X_{1} \ne \emptyset } \\ 1 & {X_{1} = \emptyset } \\ \end{array} } \right.$$

Then α – cut of A and B is in Table 2, and ${F}^{I}\left(\mathbf{a}\mathbf{l}\mathbf{m}\mathbf{o}\mathbf{s}\mathbf{t}\mathbf{a}\mathbf{l}{\mathbf{l}}_{E}\right)\left(A,B\right)$ is in Table 3.

**Table 2:** α – cut of A and B

	${\left({\varvec{A}}\right)}_{\ge {\boldsymbol{\alpha }}_{1}}$		${\left({\varvec{B}}\right)}_{\ge {\boldsymbol{\alpha }}_{2}}$
${\alpha }_{1}\in (\text{0.9,1}]$	$\{{e}_{3}\}$	${\alpha }_{2}\in [\text{1,0.8}]$	$\{{e}_{1}\}$
${\alpha }_{1}\in (\text{0.8,0.9}]$	$\{{e}_{2},{e}_{3}\}$	${\alpha }_{2}\in (\text{0.8,0.3}]$	$\{{e}_{1},{e}_{2}\}$
${\alpha }_{1}\in (\text{0.2,0.8}]$	$\{{e}_{1},{e}_{2},{e}_{3}\}$	${\alpha }_{2}\in (\text{0.3,0.1}]$	$\{{e}_{1},{e}_{2},{e}_{3}\}$
${\alpha }_{1}\in (\text{0,0.2}]$	$\{{e}_{1},{e}_{2},{e}_{3},{e}_{4}\}$	${\alpha }_{2}\in (\text{0.1,0}]$	$\{{e}_{1},{e}_{2},{e}_{3},{e}_{4}\}$

**Table 3:** $$F^{I} \left( {{\mathbf{almostall}}_{E} } \right)\left({A,B}\right)$$

${\varvec{almostall}}_{E}$	${\alpha }_{2}\in [\text{1,0.8}]$	${\alpha }_{2}\in (\text{0.8,0.3}]$	${\alpha }_{2}\in (\text{0.3,0.1}]$	${\alpha }_{2}\in (\text{0.1,0}]$
${\alpha }_{1}\in (\text{0.9,1}]$	0.02:0.00	0.05:0.00	0.02:1.00	0.01:1.00
${\alpha }_{1}\in (\text{0.8,0.9}]$	0.02:0.00	0.05:0.00	0.02:1.00	0.01:1.00
${\alpha }_{1}\in (\text{0.2,0.8}]$	0.12:0.00	0.30:0.33	0.12:1.00	0.06:1.00
${\alpha }_{1}\in (\text{0,0.2}]$	0.04:0.00	0.10:0.00	0.04:0.50	0.02:1.00

The evaluation result is calculated from Table 3 by the sum of the matrix ${F}^{I}\left({\mathbf{almostall}}_{E}\right)\left({X}_{1},{X}_{2}\right)=0.02\times 0+\dots +0.02\times 1=0.379$.

### Interpretability

The three main areas of research in fuzzy quantification are interpretation, reasoning, and summarization. The purpose of interpretation is to define the meaning of fuzzy quantification; the purpose of reasoning is to extract more information from the rules using fuzzy quantifications, and the purpose of summarization is to provide the best quantifier expression for certain situations [74]. To increase the applicability of summarization to real life, it is necessary to increase its linguistic quality by including interpretability [76]. Interpretability was studied by [77] in two ways: based on individual and group sentences. The evaluation procedure begins at the sentence level, where the quality of each sentence is assessed, quality sentences are chosen, and linguistic translation is performed. Although truth degree is typically used for evaluating this level of representation, other metrics have also been created, including Yager's informativeness level [41], Kacprzyk's quality indicators [78], and Wu and Mendel's method [79]. A summary's global interpretability depends not only on how well each sentence can be understood separately but also on how well it can be understood collectively [77]. According to the reduction algorithm for summaries [80], high-quality sentences from the resulting sentences can be found using rank-based or score-based threshold techniques. Different aspects of global interpretability are the consistency of sentences, non-redundancy, and information. A summary can be considered consistent when non-contradiction and double negation are satisfied [77]. Non-contradiction refers to two sentences with contradictory terms with complementary truth values. For the sentence *"S* = *Q B Y are A"*, two contradictory forms are *"C1(S)* = *¬Q B Y are A "* and *"C2(S)* = *Q B Y are ¬A"* where ¬ is negation. Redundancy, which happens when multiple sentences transmit the same idea and unnecessarily lengthen the summary, is the second factor in the interpretability of a summary. Non-redundancy analysis enables the removal of pointless generated sentences. First, it should be remembered that the double negation is a type of redundancy that calls for excluding either S or D(S) from the sentence. Other instances of redundancy are caused by inclusion and similarity [77]. Inclusion refers to a situation where the summarizer or quantifier of a sentence is included in the summarizer or quantifier of another. If Q ⊆ Q1 and A ⊆ A1, *“S1* = *Q B Y are A”* and *“S2* = *Q1 B Y are A1”*, then S1 is included in S2. The third aspect of summary interpretability is the knowledge that the user receives from the summary. Sentence inference and underlying meaning are two examples of information sources based on the relationships between sentences [77]. For instance, from the two summaries *"Q1 A Y are B"* and *"Q2 B Y are C"*, knowledge is *"Q Y are A and C"* where Q is the multiplication of the fuzzy numbers. The sentences *"All B Y A"* and *"All A Y C"* can be expressed in a new sentence of the form *"All B Y C"*. Suppose all *B* antecedent summarizers and *"Q B Y A"* sentences are present in the sentence set with a high degree of accuracy. In that case, they can be expressed by a single sentence *"Q Y A"*. Several sentences can be combined in terms of quantifiers and expressed in a single sentence. For example, if a set of sentences contains *"Most Y are A"*, *"Few Y are B"* and *"Few Y are C",* they can be expressed in a single sentence such as *"Y mostly are A, sometimes B and C".*

In this study, linguistic summaries from the ET and Bayley data of children with ASD and TD children are produced based on fuzzy quantifiers. With the help of semi-fuzzy-based evaluation methods defined in this section and interpretability aspects, we produced linguistic summaries and their truth degrees to describe different characteristics of children.

## Application and results

The project’s data set from [16] is used in this study. The dataset includes two groups of children: 61 young children with ASD with a mean age of 34.85 months (Range 28–36 months) and 72 TD children with a mean age of 32.90 months (Range 26–36 months) from a university-based research center in metropolitan and rural areas in Ankara, Türkiye. Children with ASD had been previously diagnosed by licensed child psychiatrists using the DSM-V criteria [1]. Children with ASD were matched with the TD group based on their chronological age since the study used a passive viewing paradigm that did not require any language processing skills. All participants had to meet specific criteria, such as being between 18 and 36 months old, not having a seizure disorder or known genetic disease, and not having an uncorrectable hearing or visual impairment. Each participant had to fulfill several requirements, including being between 18 and 36 months old, not having a genetic disease known to cause seizures, and not suffering from an untreatable hearing or vision impairment. A 17-in. screen was put beneath an SMI-Red250 [81] remote eye tracker, which recorded eye movements at a sampling rate of 250 Hz. Passive watching ET exercises were used to measure the participants' eye movements. In this study, two sets of paired preference viewing tasks were developed, and each group was given to the participants in a single session. Pairs of social and non-social stimuli make up these two groupings. The first set comprised three pairs of toy films and SI videos (social stimuli). In comparison, the second set had three animation videos and SI movies (social stimuli). This study aimed to determine whether two distinct non-social stimuli, toy or animation sets, would better capture the visual attention differences of young children with ASD than SI videos. Animation videos were evaluated as separate stimuli sets [16]. There were 14 features available in this dataset. In the SMI-Red250 manual, the definitions of the features are provided [81]. These features has been selected by using the "Attribute selection mode" of WEKA [82], an open-source ML tool, to apply the tenfold cross-validation method while extracting the distinctive features throughout the feature selection phase of the study which the dataset obtained for this study. In the feature selection, [16] identified fixation count, dwell time, and animation area of interest (AOI) features as discriminative features. Additionally, as demonstrated by approaches used in feature selection, some features, such as Net Dwell Time, are very discriminative in identifying young children with ASD. In addition to that study, Bayley scores of children have been collected. Bayley Scales, are a set of developmental assessments used to measure infants' and toddlers' cognitive, motor, and language development. These scales provide a standardized way to assess a child's developmental progress during the first few years of life [14]. The most well-known versions are the Bayley Scales of Infant Development, Third Edition (Bayley-III) [12], and the Bayley Scales of Infant and Toddler Development, Fourth Edition [83]. Our dataset recorded Bayley scores as age cognitive composite, cognitive age language, composite receptive communication, expressive communication, motor composite, fine motor, and gross motor. This study used both ET data and Bayley scores of children. The features used and their descriptions are given in Table 4 [84]. Some of the children are not included in the Bayley assessment; therefore, the combination of the ET-Bayley data set is limited to the number of children who have Bayley assessment and ET data. Consequently, 130 different children have been included in this study.

**Table 4:** Features

Feature	Description	Value
Net Dwell Time	The time fixated on a particular point	< low,medium,high >
Dwell Time	The total amount of time a participant fixates	< low,medium,high >
Glance duration	The time the gaze moves towards a target	< low,medium,high >
Diversion duration	The sum of the diversion duration of all subjects divided by the number of the subjects	< low,medium,high >
First fixation duration	The time that the first fixation lasted	< low,medium,high >
Fixation count	Number of all fixations for selected subjects	< low,medium,high >
Fixation time	The time of fixation	< low,medium,high >
Cognitive composite	The child's overall cognitive development score	< low,medium,high >
Cognitive age	Level of cognitive development demonstrated by a child compared to a typical age range	< far behind, behind,equal,ahead,far ahead >
Language composite	The child's overall language development score	< low,medium,high >
Receptive communication	The child's ability to understand and comprehend language	< far behind, behind,equal,ahead,far ahead >
Expressive communication	The child's ability to communicate using words, gestures, or other means	< far behind, behind,equal,ahead,far ahead >
Motor composite	The child's overall motor development	< low,medium,high >
Fine motor	Coordination and control of small muscles in the hands and fingers	< far behind, behind,equal,ahead,far ahead >

### Application

**Fig. 2:**
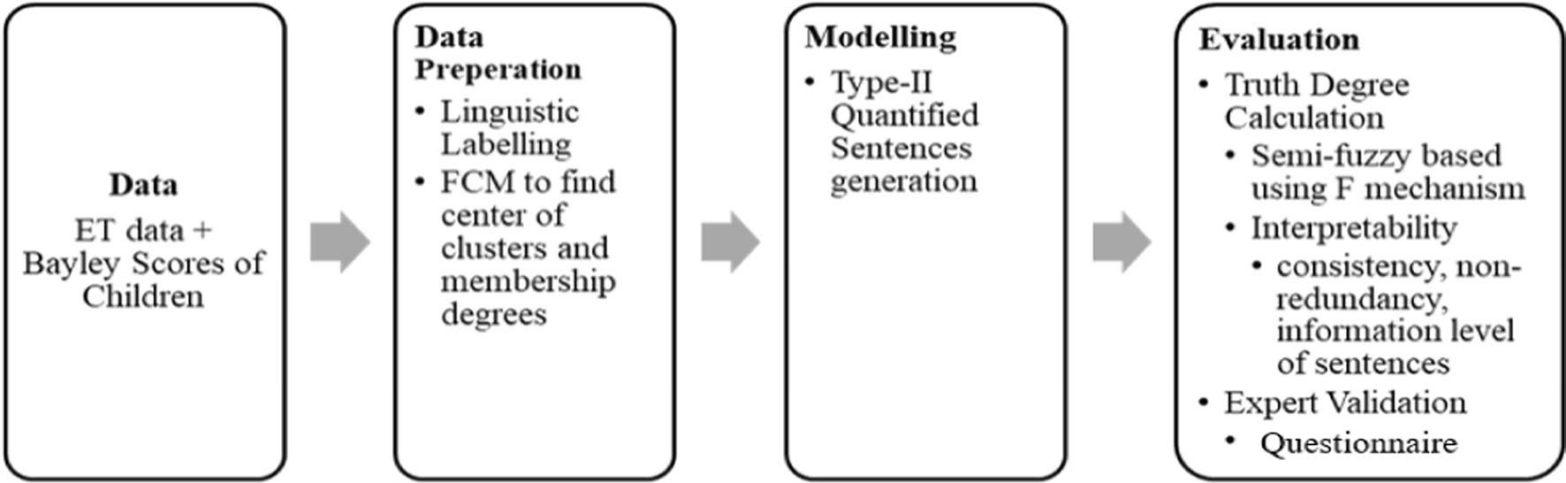
Process of the application

The application of fuzzy linguistic summarization of ET data of children when they are watching animation-SI set addition to their Bayley scores is based on semi-fuzzy sentences. Semi-fuzzy sentences allow us to produce informative sentences to compare ET and Bayley features of children. The application process is given in Fig. 2. The application started by creating a dataset that combines ET data and Bayley scales of children. In the data preparation phase, linguistic labels are defined, and FCM is applied to find the centers of each linguistically labelled fuzzy set. In the modelling and evaluation phases, type-II quantified sentences are created, and their truth degrees are calculated based on the semi-fuzzy quantifier-based evaluation method. After the generated summaries are revised according to the interpretability of the linguistic summaries, they are then presented to experts for validation. With this process, we have extended the well-known linguistic summarization method to the ASD screening area and summarized the features that affect the classification of ASD between children.

**Fig. 3:**
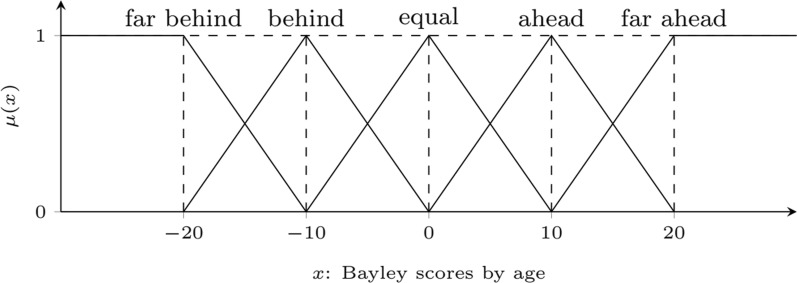
Sets of Bayley scores

**Fig. 4:**
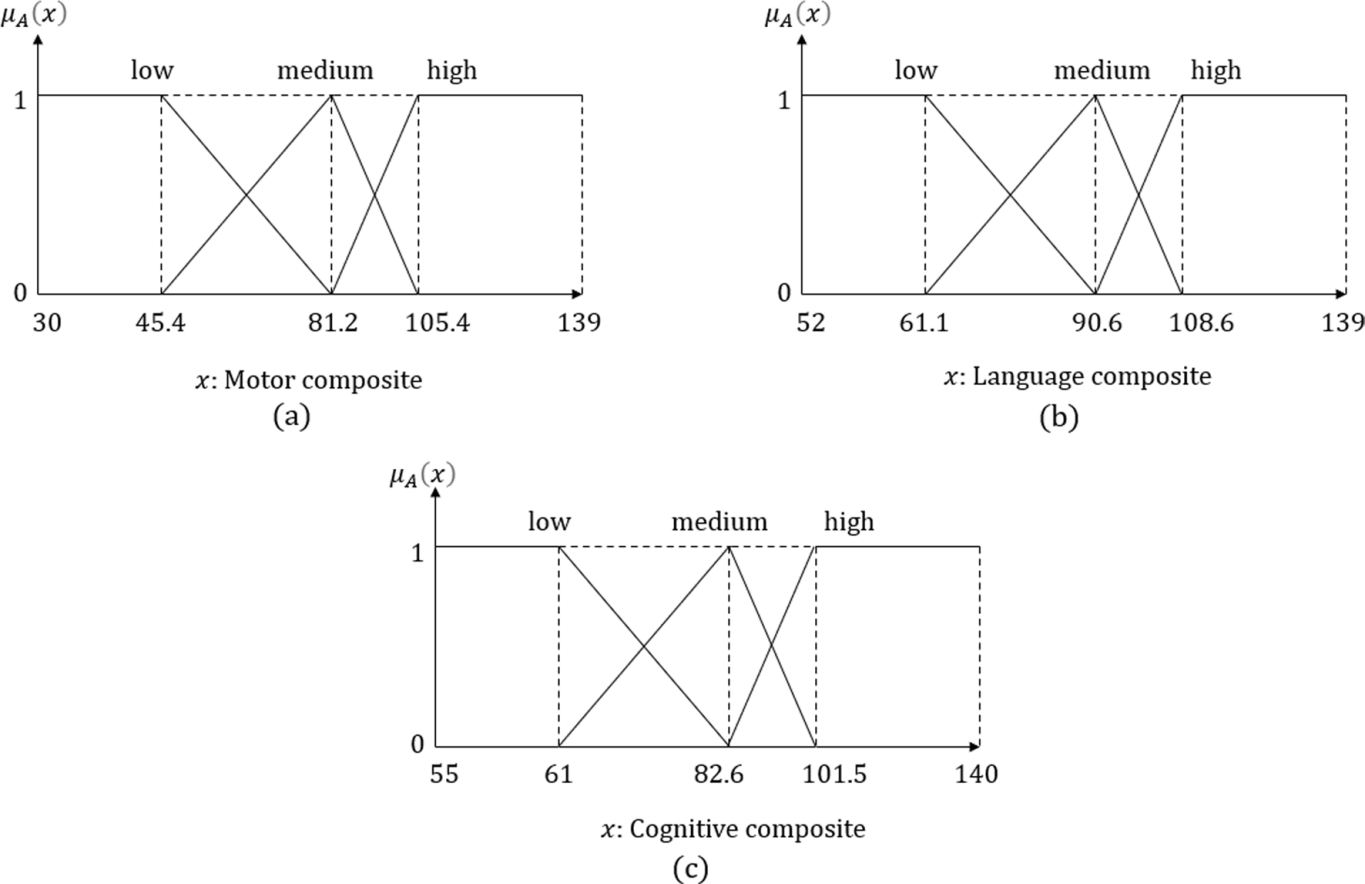
The fuzzy sets of composite features

The features of the Animation-SI set used in the generation of sentences are selected according to features suited as discriminative based on feature selection methods [16]. These features are net dwell time, dwell time, glance duration, diversion duration, first fixation duration, fixation count, and fixation time, which belong to SI and animation visual attention of children. Composite features of Bayley data of children using FCM were divided into three fuzzy sets: low, medium, and high. Other features have been divided into five groups according to their age "behind by age", "far behind by age", "equal to age", "ahead by age", "far ahead by age" and given in Fig. 3. The fuzzy sets of composite features are presented in Fig. 4.

The Bayley dataset also includes the age and cognitive age of children. Based on these features, children are evaluated by always comparing their feature values according to their age. Therefore, the children can be evaluated as "ahead by cognitive age" or "receptive communication is behind of the age". ET features have been divided into three fuzzy sets: low, medium, and high with the FCM algorithm. Few, about half, and most are the quantifiers employed in the sentences.

**Fig. 5:**
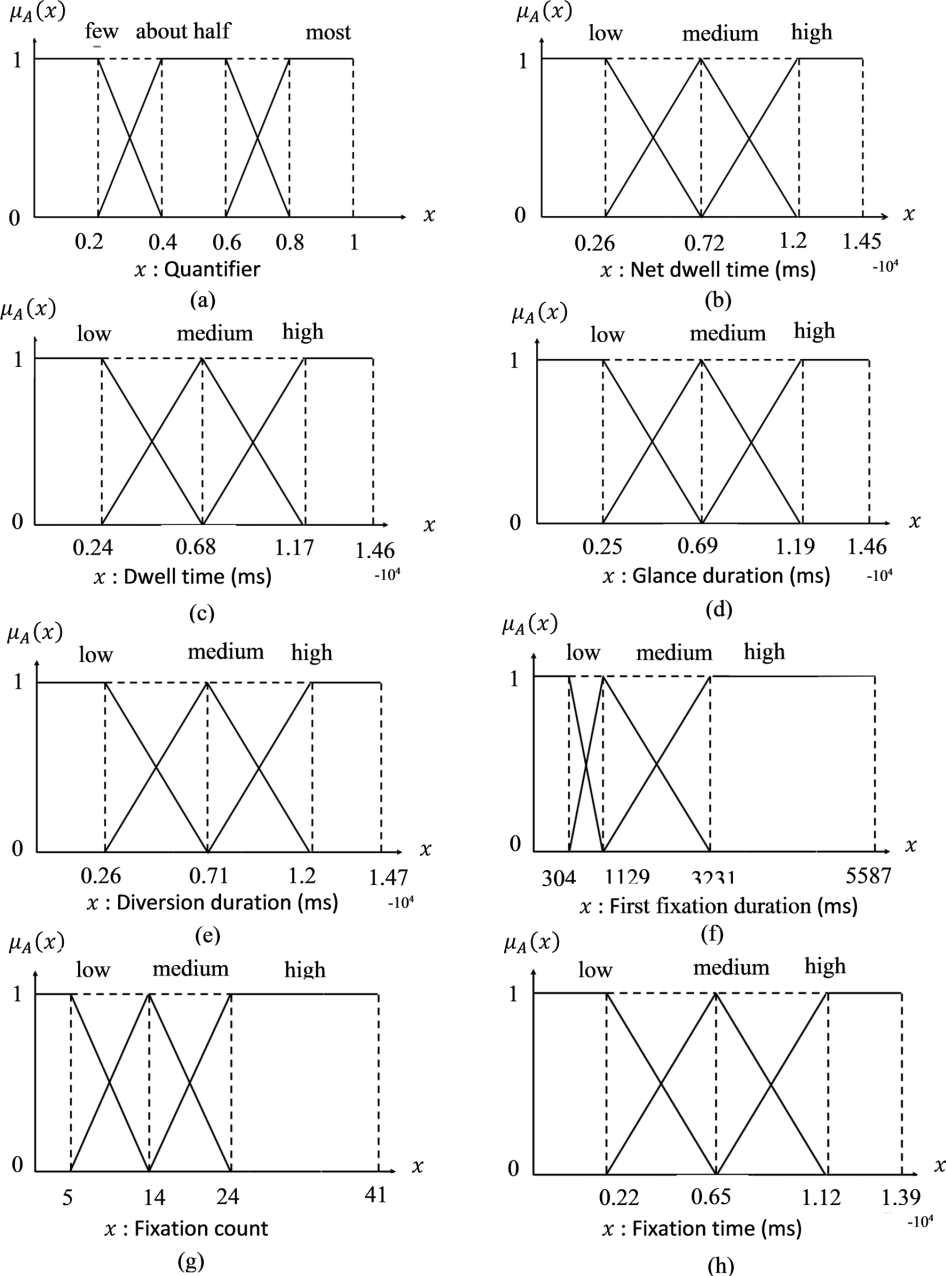
**a** Linguistic quantifier and fuzzy sets of numerical variables **b** net dwell time, **c** dwell time, **d** glance duration, **e** diversion duration, **f** first fixation duration, **g** fixation count and **h** fixation time

Fuzzy sets for ET features and quantifiers are given in Fig. 5. All the combinations of summarizers, pre-summarizers, and quantifiers were generated by MATLAB [85].

Because of the use of semi-fuzzy quantifier-based truth degree calculation for linguistic summaries, the ${F}^{I}$ mechanism was used, and sentence evaluation was done by selecting sentences based on the threshold where the truth degree is larger than 0.90. Some of the selected quantified sentences and the truth degrees are given in Table 5. In this table, the sentences with the "most" quantifier are suited, because the sentences with quantifier "few" are their negations.

**Table 5:** Generated sentences for children with ASD and TD children

Linguistic summary	T
Most of the ASD child who is far behind by cognitive age also has low language composite	0.95
Most of the ASD child who is far behind by cognitive age also has receptive communication far behind by age	0.9
Most of the ASD child who is far behind by cognitive age also has expressive communication far behind by age	0.89
Most of the ASD child who has gross motor far behind by age also has receptive communication far behind by age	0.98
Most of the ASD child who has high SI diversion duration also has low animation net dwell time	0.92
Most of the ASD child who has high SI diversion also has high SI net dwell time	0.82
Most of the ASD child who has low cognitive composite also has low language composite	0.92
Most of the ASD child who has high SI net dwell time also has low animation net dwell time	0.93
Most of the TD child who has low animation entry time also has low animation fixation count	0.91
Most of the TD child who has expressive communication behind by age also has low animation fixation count	0.96

Study results showed that ASD children have a passive visual attention preference for animation videos instead of SI videos. The majority of TD children preferred to watch SI videos and demonstrated enhanced visual attention towards SI videos. Therefore, our findings indicated that although TD children chose SI videos, children with ASD preferred animation videos. These findings emphasize the significance of comprehending the visual attentional differences between children with ASD and TD children, as well as how these differences may influence the children's preferences for particular genres of videos. By considering sentences that include Bayley scale features of children, it is understandable that if the children with ASD are behind by age, their communication features are also behind by age. If their cognitive composite is low, their language composite is also low. It shows that children with ASD produce coherent results in their cognitive assessments, but they are different from TD children. There are also sentences that indicate the similarity between children with ASD and TD children. It is apparent that the combination of all features for children with ASD and TD separately generates too many sentences. Revealing the differences between children by reading too many sentences takes time and effort. Therefore, the techniques for summary interpretation have been used to create sentences that can be understood collectively. First, high-quality sentences from the resulting set of sentences, which have been generated for ASD and TD groups, have been selected. If the sentences are present in the sentence set with high accuracy, they have been expressed by a single sentence. Then, several sentences have been combined in terms of quantifiers and expressed in a single sentence instead of using two different sentences for ASD and TD. Furthermore, considering the consistency of sentences and the information that sentences provide, the sentences refer to the same idea and unnecessarily lengthen the summary; pointless generated sentences have been removed. Thus, the results fulfilled non-redundancy, and the linguistic quality of the sentences was increased. The resulting linguistic summaries are given in Table 6 for children with ASD and Table 7 for TD children.

**Table 6:** Linguistic summaries for children with ASD

Linguistic summary	T
Most of the children with ASD who are far behind by age or whose gross motor skills are far behind by age have low SI average fixation duration and low language composite, and their receptive communication is far behind by age	0.90
Most of the children with ASD who have high animation net dwell time or high animation fixation count also have low SI net dwell time, dwell time, glance duration, diversion, fixation time, average fixation duration	0.90
Most of the children with ASD whose receptive communication is far behind by age have low language composite, and their expressive communication is also far behind by age	0.90
Most of the children with ASD who have high SI net dwell time have low animation net dwell time, fixation count, fixation time	0.90
Most of the children with ASD who have high animation dwell time have low SI fixation time and average fixation duration	0.90
Most of the children with ASD whose expressive communication is far behind by age have low language composite and their receptive communication is far behind by age	0.96
Most of the children with ASD whose fine motor is far behind by age also have low language composite and their receptive communication is far behind by age	0.92

**Table 7:** Linguistic summaries for TD children

Linguistic summaries	T
Most of the TD children with high animation entry time also have low animation net dwell time, dwell time, glance duration, diversion, glances count, revisit, fixation count, and fixation time	0.96
Most of the TD children who have low animation diversion also have low animation fixation count and fixation time	0.95
Most of the TD children who have high SI diversion duration also has low animation net dwell time, dwell time, glance duration	0.95
Most of the TD children who have high SI fixation count or SI glance duration also have low animation net dwell time, dwell time, glance duration, diversion, fixation count, fixation time	0.94
Most of the TD children whose expressive communication is ahead by age have high cognitive, language and motor composite	0.91
Most of the TD children who have low animation net dwell time or low animation fixation time or low glances count have low animation dwell time, glance duration, fixation count, fixation time	0.93
Most of the TD children who have high SI net dwell time have low animation dwell time, glance duration, fixation count, fixation time, dwell time, diversion	0.95
Most of the TD children who have low animation dwell time have low animation net dwell time, glance duration, fixation count, fixation time, diversion	0.95
Most of the TD children who have high SI fixation time or low animation revisit have low animation net dwell time, glance duration, fixation count, fixation time, dwell time, diversion	0.95
Most of the TD children who have low SI glances count have low animation glance duration, diversion, revisit, fixation count, fixation time	0.99
Most of the TD children who have high SI dwell time have low animation dwell time, glance duration, fixation count, fixation time, net dwell time, diversion	0.95
Most of the TD children who are ahead by cognitive age have low animation fixation count and they have high cognitive composite	0.90
Most of the TD children who have low animation diversion also have low animation net dwell time, dwell time, glance duration, diversion	0.95
Most of the TD children who have low SI revisit also have low animation net dwell time, dwell time, glance duration, diversion, glances count, fixation count, fixation time	0.98

It is seen that for children with ASD, their communication or motor skills are compatible with each other. Especially if the children with ASD are behind or far behind by age in cognitive score, fine or gross motor, language or expressive communication, and language composite scores are the discriminative features. These cognitive composite features differ between children with ASD and TD, as shown in the generated summaries. These sentences don’t indicate that children with ASD’s fixation duration is related to their language composite, receptive communication, or gross motor scores. On the other hand, children with ASD pay more attention to animation videos than SI videos. Furthermore, it can be revealed from the summarizations that net dwell time is a discriminative feature, which is also supported by the study [16].

Furthermore, it is seen that if the animation entry time results are high animation net dwell time, dwell time, glance duration, diversion, glances count, revisit, fixation count and fixation time for TD children are low or vice versa. It reveals that these ET features differ from the children with ASD and TD children. Furthermore, composite features are also high for children if expressive communication is high for children. Net dwell time in animation videos is also a discriminative feature for TD children. While SI glance count and SI revisit time are high, animation glance duration, diversion, revisit, fixation count, and fixation time are high. If SI dwell time is high, animation-related features are low. On the other hand, findings revealed that the cognitive age of TD children was related to their cognitive composite scales.

This study was conducted to reveal the differences between children with ASD and TD children in natural language. For this purpose, ET data were collected from children with ASD and TD children. In addition, Bayley scales used in children's assessment were also added to this data set. Linguistic summaries were produced with the obtained data set, and linguistic summaries with a high degree of truth were selected. The obtained linguistic summaries revealed the monitoring tendencies and cognitive characteristics of children with ASD and their differences from those of TD children. For example, there are two summaries: most of the TD children who have high SI net dwell time have low animation dwell time and most of the children with ASD who have high animation net dwell time have low SI net dwell time. These sentences show that net dwell time is a distinguishing feature between children with ASD and TD children, and children with ASD prefer to watch animation video instead of SI video. Therefore, the animation net dwell time value may help indicate ASD among children. Explaining these differences with natural language has become a resource for clinical studies of experts in autism. In addition, it has formed a basis for autism prediction studies with similar data sets in terms of the understandability of the subject and the ET data set and Bayley scales. Linguistic summarization plays a significant role in improving the screening and assessment of ASD. The application of linguistic summarization has a substantial role in the understanding of screening and assessment of ASD. It is a pioneering study that has the potential to help the assessment of ASD for clinicians as well as individuals who need to understand the differences between ASD and TD children, such as families or researchers. Early detection of ASD is crucial for early intervention and support. Linguistic summarization is used to analyze patterns in children's characteristics and help identify differences in children at a very young age, which may indicate the need for further evaluation. It is understood from this application that linguistic summarization allows for quantifying differences between ASD and TD children. This means researchers, clinicians, or non-expert individuals can measure the degree of variation in features, providing a more precise understanding of the development differences in children with ASD and TD. While this study's linguistic analysis focuses on differences, it can also highlight areas of similarity between ASD and TD children. Integrating linguistic summary into ASD screening is a new and noteworthy development that offers possible advantages and new insights for people with ASD, their families, and healthcare professionals.

Knowing that the quality of the linguistic summaries is determined by truth degree calculation, the validation of these summaries by an expert also helps verify the effectiveness and quality of the summary. Based on the approach in [86] a survey was conducted on two experts on ASD. The summaries in Tables 6 and 7 were presented to experts, and the following five questions were asked for validation. Total number of summaries in these tables are 21.

Question-1: What is the informative level of the summaries?

Question-2: How accurately do the summaries express the differences between children with ASD and TD?

Question-3: How simple and understandable are the summaries?

Question-4: At what level is the content richness of the summaries?

Question-5: How useful is it to have summaries based on general and comparative characteristics?

Experts were asked to evaluate the questions on a scale of 1–10 (1 "very negative" to 10 "very positive"). The results were calculated according to the formulas in Eqs. (15) and (16)

15 $$Q_{{S_{i} }} = \frac{{\frac{{{ }\overline{P1} + { }\overline{P2} }}{2} + \frac{{{ }\overline{{P3 + { }\overline{P4} + { }\overline{P5} }} }}{3} }}{2}$$

16 $$GQ = \mathop \sum \limits_{i = 1}^{n} \frac{{Q_{{S_{i} }} }}{n}$$

The terms $\overline{P1 }$, $\overline{P2 }$, $\overline{P3 }$, $\overline{P4 }$, $\overline{P5 }$ are the average of the answers received from experts to questions 1, 2, 3, 4 and 5, respectively. Thus, the global quality score ($GQ$) for generated summaries is obtained as the average of the validation. ${Q}_{{S}_{i}}$ is defined as the arithmetic mean of the two dimensions where questions 1 and 2 considered for truthfulness and relevance and questions 3,4, and 5 considered to assess how the summaries are well-organized and clear. The number of summaries presented to experts (n) is 21. According to these values, $\overline{P1 }$, $\overline{P2 }$, $\overline{P3 }$, $\overline{P4 }$, $\overline{P5 }$ are 9, 8.5, 9, 9, 10 respectively. The summaries' global quality score was 9.05 out of 10. This score indicates that the summaries’ quality is high enough.

## Limitations and future directions

Currently, behavioral, historical, parent-report, and interview assessments—all of which are subjective, labor-intensive, and time-consuming—are the primary tools used to diagnose ASD. The screening and diagnosis of ASD are limited by the absence of objective methods for assessment [11]. Traditional methods of diagnosing ASD include behavioural observations, historical records, parental reports, and statistical analysis [87]. Eye-tracking systems, which record gaze patterns such as gaze fixation, blinking, and saccade eye movements, are examples of advanced technologies that can be employed. Given this capacity, a significant contribution can be achieved by creating a model intended to investigate the differences in gaze patterns and attention mechanisms between children diagnosed with ASD and children who have not [88]. This linguistic summarization study is the first pioneering work in a promising field that can provide convenience, especially to people who are not experts in the field and help clinicians. Linguistic summarization of ET and Bayley data of children is a significant tool for understanding child development, but limitations and challenges exist. ET data and Bayley scores can be variable; therefore, data collection from different children is also crucial regarding data quality for sufficient results. Age-related differences in data need to be considered when interpreting data. Bayley assessments are age-appropriate and sensitive to developmental changes [89]. Due to that, the preparation of the Bayley data for linguistic summarization or any other data-driven approaches requires specific domain knowledge. Because of the difficulties in data collection from children within the same or near age, access to large datasets of ET and Bayley data from children can be limited. Despite limitations, the collection of more extensive and diverse ET data sets encompassing a wide range of ages, integrating multiple data like EEG or other assessments, including disciplines in developing and validating linguistic summarization techniques, can enhance the outcome. Linguistic summaries can be applied across different cultural contexts. This allows for examining linguistic development in diverse populations, potentially revealing universal and culture-specific patterns. Linguistic summarization techniques can become more effective tools for understanding and supporting children with ASD and TD. Preparing raw data for additional processing and analysis is known as data preparation. This issue becomes more important as choosing the right features in large and complex data sets increases the effectiveness of the results. Especially in health systems, the outcomes can be critical in diagnosis or treatment. Hence, heuristic optimization algorithms such as Binary Gray Wolf Optimization, Binary Genetic Algorithm [90], Geyser Inspired Algorithm [91], Dwarf Mongoose Optimization Algorithm [92], and Genghis Khan shark optimizer [93] can be used to select the most significant set of features or to eliminate invalid data. In addition, linguistic summarization consists of generating all summaries, calculating the degree of truth of the summaries, and selecting summaries with high accuracy. Since the number of summaries produced is very high and all summary combinations must be evaluated mutually to select the summaries above a threshold, the study can be transformed into an optimization problem for selecting summaries under constraints, and a more efficient search of the universal set can be achieved with meta-heuristic algorithms mentioned above. Therefore, considering these challenges and possibilities, using different linguistic summarization techniques on the ET-Bayley data set to improve the dataset's variety and/or volume is suggested.

## Conclusion

Linguistic summarization in this study uses the integration of multiple data sources. ET data provide insights into visual attention and gaze patterns, while Bayley data assess developmental milestones. Therefore, it allows us to examine the relationship between visual attention and developmental scores in young children with ASD by interpreting the significance of ET patterns concerning the general development of children. Integrating ET with Bayley data allows for the tracking and identifying critical deviations from TD children. Therefore, clinicians or experts can also use linguistic summaries in their assessments and interventions. Linguistic summarization is an essential tool with ET and Bayley data, enhancing our ability to analyze and interpret data in children with ASD and TD. It clarifies the complex relationship between visual attention, language development, and cognitive skills, ultimately advancing the diagnosis and our comprehension of child development. Moreover, understanding a child's specific profile through linguistic summaries allows for developing personalized interventions. ET data has recently been widely used in ASD screening. In addition, Bayley scales have been used in ASD assessment for many years. Interpretation of these two data can only be done by experts. Both data are used to observe and diagnose differences between children with ASD and TD. The advantages of this study are that it demonstrated the differences between ASD and TD children with summary sentences using these two data and created a basic understanding for studies that will use these data. It also facilitated non-experts' understanding of ET data and Bayley scales. Linguistic summarization is a pioneering work in ASD diagnosis and shows promise in overcoming the difficulties encountered in the diagnosis of ASD between children by making features of ET behaviors of children and their Bayley scores easy to understand and interpret. It can also be used as a basis for classification-based prediction studies of ASD assessment.

In conclusion, the linguistic summarization represents a transformative step forward in elucidating the differences between ASD and TD. This innovative approach stands as the first study dedicated to unravelling the intricate relationship between ET data and Bayley scales in ASD screening. Through the process of collapsing complex datasets into easily understandable insights about visual attention patterns and cognitive processes, this method enables researchers and practitioners to get deep insights. Better results for those on the autism spectrum may result from more focused and effective interventions, which are made possible by this growing understanding. Furthermore, through the promotion of early ASD assessment and a more nuanced understanding of the distinctive traits shared by children with ASD and TD, linguistic summarization acts as a stimulant for the development of inclusivity, empathy, and customized support for children with neurological disorders at different stages of their development.

## Data Availability

Not applicable.
